# Transporter Protein-Guided Genome Mining for Head-to-Tail Cyclized Bacteriocins

**DOI:** 10.3390/molecules26237218

**Published:** 2021-11-28

**Authors:** Daniel Major, Lara Flanzbaum, Leah Lussier, Carly Davies, Kristian Mark P. Caldo, Jeella Z. Acedo

**Affiliations:** 1 Department of Biology, Mount Royal University, Calgary, AB T3E 6K6, Canada; dmajo818@mtroyal.ca (D.M.); lflan365@mtroyal.ca (L.F.); cdavi671@mtroyal.ca (C.D.); 2 Department of Chemistry and Physics, Mount Royal University, Calgary, AB T3E 6K6, Canada; lluss039@mtroyal.ca; 3 Department of Agriculture, Food and Nutritional Science, University of Alberta, Edmonton, AB T6G 2P5, Canada; caldo@ualberta.ca

**Keywords:** antimicrobials, head-to-tail cyclized bacteriocins, circular bacteriocins, genome mining

## Abstract

Head-to-tail cyclized bacteriocins are ribosomally synthesized antimicrobial peptides that are defined by peptide backbone cyclization involving the N- and C- terminal amino acids. Their cyclic nature and overall three-dimensional fold confer superior stability against extreme pH and temperature conditions, and protease degradation. Most of the characterized head-to-tail cyclized bacteriocins were discovered through a traditional approach that involved the screening of bacterial isolates for antimicrobial activity and subsequent isolation and characterization of the active molecule. In this study, we performed genome mining using transporter protein sequences associated with experimentally validated head-to-tail cyclized bacteriocins as driver sequences to search for novel bacteriocins. Biosynthetic gene cluster analysis was then performed to select the high probability functional gene clusters. A total of 387 producer strains that encode putative head-to-tail cyclized bacteriocins were identified. Sequence and phylogenetic analyses revealed that this class of bacteriocins is more diverse than previously thought. Furthermore, our genome mining strategy captured hits that were not identified in precursor-based bioprospecting, showcasing the utility of this approach to expanding the repertoire of head-to-tail cyclized bacteriocins. This work sets the stage for future isolation of novel head-to-tail cyclized bacteriocins to serve as possible alternatives to traditional antibiotics and potentially help address the increasing threat posed by resistant pathogens.

## 1. Introduction

Bacteriocins refer to a highly diverse family of bacteria-derived ribosomally synthesized antimicrobial peptides [1]. They are mainly used as biopreservatives in food and animal industries and are utilized by adding the bacteriocin-producing organism, bacteriocin-containing fermentates, or purified peptides into food, animal feed, and other antimicrobial products [1,2,3]. Recent studies have shown that bacteriocins also play a crucial role in regulating gut microbiota [4,5].

A class of bacteriocins referred to as head-to-tail cyclized bacteriocins are characterized by a cyclic peptide backbone wherein an amide bond is formed between the N- to C-terminal amino acids [6,7,8]. Moreover, experimentally characterized head-to-tail cyclized bacteriocins display a saposin-fold composed of four to five helices in a compact globular architecture that is also observed for leaderless bacteriocins [9,10]. The compact saposin-fold and cyclic peptide backbone of head-to-tail cyclized bacteriocins confer increased stability against proteolytic digestion and denaturation typically caused by extreme temperature and pH conditions [9]. There are two suggested subgroups of head-to-tail cyclized bacteriocins. Subgroup i head-to-tail cyclized bacteriocins are highly cationic resulting in high (generally >10) isoelectric point values, while subgroup ii bacteriocins are mainly neutral and have lower isoelectric points [6,8].

Several head-to-tail cyclized bacteriocins are active against a wide range of Gram-positive bacteria while activity against Gram-negative strains was observed when the bacterial membrane integrity was initially compromised with ethylenediaminetetraacetic acid (EDTA) or with the use of higher bacteriocin concentrations [7,11,12,13,14]. These antimicrobial peptides are reported to operate via diverse mechanisms of action that mostly involve pore formation in the target lipid membranes [15,16,17]. The most studied head-to-tail cyclized bacteriocin, enterocin AS-48, forms a dimer that non-specifically creates toroidal pores in lipid membranes [18,19]. Another head-to-tail cyclized bacteriocin, carnocyclin A, was reported to create anion-selective channels as a monomeric unit [16,20]. More recently, garvicin ML was shown to bind to a maltose ABC transporter complex that mediates activity particularly at low bacteriocin concentration [21].

Genes encoding proteins that are involved in the biosynthesis of head-to-tail cyclized bacteriocins are clustered together (i.e., in a biosynthetic gene cluster) either in the chromosomal DNA or plasmid DNA of the producer organism [7]. A typical biosynthetic gene cluster for head-to-tail cyclized bacteriocins consists of genes encoding the bacteriocin precursor peptide, transporter protein(s), a SpoIIM (stage II sporulation protein M) membrane protein (previously known as DUF95), an immunity protein, and one or more unknown hydrophobic proteins [6,7,8]. The inactive precursor peptide has an N-terminal leader sequence and C-terminal core peptide. During maturation, the leader peptide is cleaved, and a peptide bond is formed between the new N-terminal amino acid and the C-terminal residue, producing the active head-to-tail cyclized bacteriocin. The detailed mechanism by which this post-translational modification occurs has yet to be determined [7].

With the rapid advances in DNA sequencing technologies and the development of bioinformatic tools, a plethora of genomic data can now be mined for biosynthetic gene clusters that potentially encode novel natural products, both ribosomal and non-ribosomal in origin [22,23,24,25,26,27]. In 2017, the head-to-tail cyclized bacteriocin pumilarin from *Bacillus pumilus* B4107 was discovered through genome mining with the use of the bioinformatics tool BAGEL3 wherein the authors filtered their candidate biosynthetic gene clusters based on the presence of genes encoding a precursor peptide and a SpoIIM protein [28,29]. More recently, cerecyclin from *Bacillus cereus* DDD103 was identified through BLAST analysis using the precursor peptide sequences of known head-to-tail cyclized bacteriocins as search queries [30].

To further expand the repertoire of head-to-tail cyclized bacteriocins, we adopted a new genome mining strategy to search for novel head-to-tail cyclized bacteriocins. In particular, transporter protein sequences associated with 19 reported head-to-tail cyclized bacteriocins were used as query sequences in mining for putative head-to-tail cyclized bacteriocins. The Rapid ORF Description and Evaluation Online Tool (RODEO) was then employed to investigate neighboring genes and identify high probability functional biosynthetic gene clusters [31]. A total of 387 strains that potentially encode putative head-to-tail cyclized bacteriocins were identified, 127 of which are unique sequences. More than a hundred hits (i.e., 366 strains; Table S1) identified in this study were not detected in a previous precursor peptide-based genome mining study [32], highlighting the relevance of a transporter protein-based genome mining strategy to expand the collection of this family of circular antimicrobials.

## 2. Results and Discussion

### 2.1. Identification of Putative Head-to-Tail Cyclized Bacteriocins

The transporter protein sequences associated with characterized head-to-tail cyclized bacteriocins were used as driver sequences to search the NCBI genome database of non-redundant protein sequences (accessed 30 June 2020) for novel head-to-tail cyclized bacteriocins. The genomic sequences of the protein hits were then analyzed to determine which transporter proteins were clustered with genes encoding a bacteriocin precursor peptide and a SpoIIM protein, since previous studies have shown that biosynthetic gene clusters of experimentally characterized head-to-tail cyclized bacteriocins contain at least these three genes (i.e., precursor peptide, SpoIIM, and transporter protein) [7,30,32]. In total, 387 hits were detected, 127 of which are unique peptide sequences (Table S1).

Several precursor peptides are encoded in multiple different organisms, while ~25% of the peptides are exclusively found in a single organism (Table S1). For example, the biosynthetic gene clusters for the characterized bacteriocins, amylocyclicin and pumilarin, are found in 61 and 43 different strains, respectively. On the other hand, carnocyclin A, aureocyclicin 4186, and garvicin ML, are produced exclusively by *Carnobacterium maltaromaticum* UAL307, *Staphylococcus aureus* 4185, and *Lactococcus garvieae* DCC43, respectively (Table S1). 

**Figure 1:**
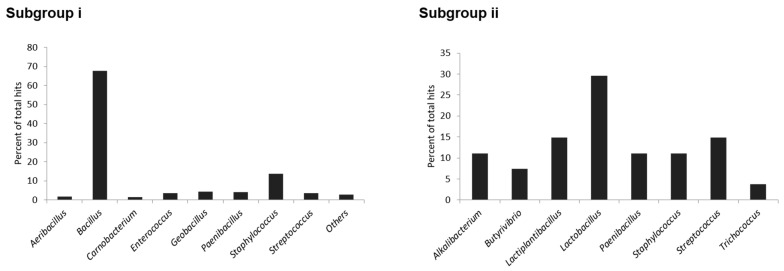
Genera distribution of subgroup **i** and subgroup **ii** head-to-tail cyclized bacteriocin producer organisms identified in this study.

The producer organisms of head-to-tail cyclized bacteriocins identified in this study span various bacterial genera, which are mainly under the Firmicutes phylum (Figure 1). The members of each subgroup are differentially distributed across different genera. Subgroup i head-to-tail cyclized bacteriocins are predominantly found in *Bacillus* followed by *Staphylococcus* and *Geobacillus*. The *Bacillus* genus is regarded as a gold mine of antibiotic candidates, not only for bacteriocins, but for lipopeptide antibiotics as well [33,34,35,36,37].

Subgroup ii members were identified mainly in *Lactobacillus*, *Lactiplantibacillus*, and *Streptococcus* bacteria. *Paenibacillus*, *Staphylococcus* and *Streptococcus* were found to encode for biosynthetic genes for the two head-to-tail cyclized bacteriocin subgroups, whereas the other genera only encode genes from either one of the two subgroups.

### 2.2. Amino Acid Sequence Diversity of Identified Precursor Peptides

**Figure 2:**
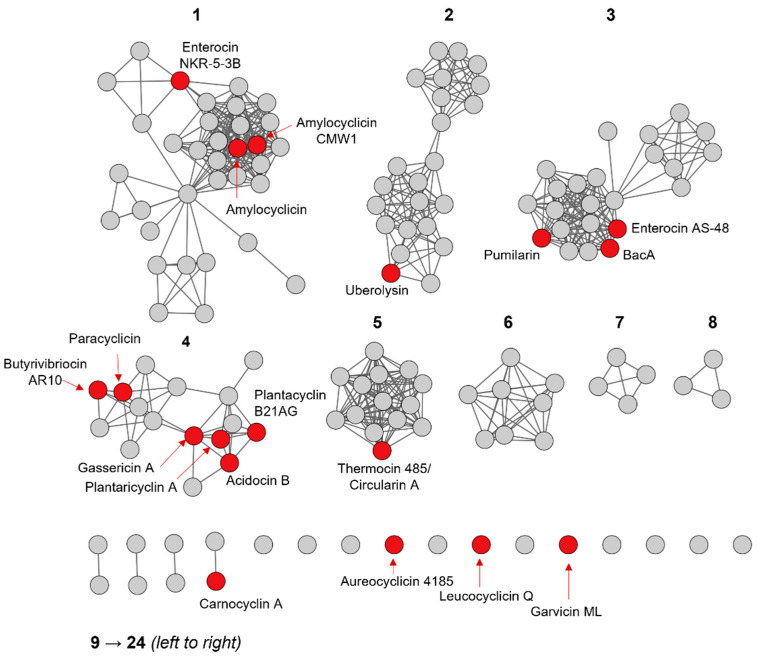
Amino acid sequence similarity network of head-to-tail cyclized bacteriocin precursor peptides resulting in 12 groups and 12 singletons. The group numbers are indicated in bold. Putative novel head-to-tail cyclized bacteriocins are shown in gray, while characterized bacteriocins are shown in red and are labelled.

To investigate the diversity of the identified putative head-to-tail cyclized precursor peptide sequences, an all-by-all BLAST analysis was performed to create a sequence similarity network that grouped together the most related peptides (Figure 2). The results were then visualized using the Cytoscape program (version 3.9) [38]. In the sequence similarity network (Figure 2), each circle, referred to as a node, represents a unique peptide sequence. A line, referred to as an edge, is drawn between two nodes that share a similarity within the defined alignment score threshold.

The sequence similarity network analysis resulted in twelve groups and twelve singletons (Figure 2). The largest group is comprised of 34 unique peptide sequences that are produced by a total of 119 different bacterial strains (Table S1). Characterized members of this group include amylocyclicin, amylocyclicin CMW1, and enterocin NKR-5-3B. As mentioned earlier, amylocyclicin itself is produced by 61 different bacterial strains, and hence comprises ~50% of the organisms in this group. Amylocyclicin CMW1 is found exclusively in *Bacillus amyloliquefaciens* CMW1, while enterocin NKR-5-3B is produced by four different *Enterococcus* strains. Group 2 is comprised of 23 peptides produced by 39 different strains and are closely related to the characterized bacteriocin, uberolysin. The latter was detected in genomes of 13 different organisms. Group 3 has 3 characterized members (i.e., enterocin AS-48, pumilarin, and BacA) and 20 putative members. The sequences within this group are produced by 114 different strains, signifying that several members of this group are present in multiple organisms, including the characterized bacteriocin, pumilarin, which is specifically found in 43 different strains.

Interestingly, all the previously classified as subgroup ii head-to-tail cyclized bacteriocins (i.e., paracyclicin, butyrovibriocin AR10, acidocin B, gassericin A, plantaricyclin A, and plantacyclin B21AG) are found in a single group (group 4). This suggests that head-to-tail cyclized bacteriocins are more diverse than previously thought in terms of sequence homology and properties, and may likely not be restricted to the earlier proposed two subgroups. Subgroup i head-to-tail cyclized bacteriocins are defined by their cationic character and relatively high isoelectric point values; while subgroup ii bacteriocins are distinguished by their hydrophobicity, neutral property, and lower isoelectric points [6,8]. This classification scheme is in fact related to the amino acid sequence because the hydrophobic and electrostatic properties of peptides are dictated by the amino acid composition. This highlights the importance of the sequence similarity network presented in this work that revealed a more diverse classification scheme for head-to-tail cyclized bacteriocins. It is also worth noting that several of the uncharacterized members of group 4 are produced by *Streptococcus pneumoniae* that differ at either the strain or sub strain level. *S. pneumoniae* strains are reported to produce a major family of bacteriocins known as pneumocins, including a potential circular bacteriocin provisionally named pneumocyclicin, which was found to be present in 34% of pneumococcal genomes [39]. Interestingly, all pneumococci express a pneumocin-capable ABC transporter ComAB, but only 25% express the more efficient ABC transporter BlpAB, suggesting there is diversity in how pneumocins are used by different strains [40]. Because *S. pneumoniae* are opportunistic pathogens of global renown, understanding how their bacteriocin production confers a competitive growth advantage in the nasopharyngeal environment could be greatly beneficial to medicine.

Group 5 includes the characterized bacteriocins, thermocin 485 and circularin A, which are identical peptides. Group 5 also has 13 putative bacteriocin members. Groups 6 to 11 do not have any characterized members, and thus, further investigation of these groups via isolation and characterization is desired. Group 7 consists of 4 unique peptide sequences that are found in a total of 43 strains. Each of these peptides come from the genus *Staphylococcus* and varies by species and strain, although the majority are from *Staphylococcus aureus* (Table S1). It is a promising group to investigate because none of the peptides are very well annotated on GenBank. *S. aureus* is another clinically relevant bacterium because some strains have developed methicillin resistance (MRSA). Understanding bacteriocin synthesis may be helpful for controlling these populations in pathogenic contexts.

Group 12 is comprised of two unique peptide sequences, one of which is the well-characterized bacteriocin, carnocyclin A. Lastly, among the 12 singletons (which share the lowest similarity to the rest of the identified peptides) are the experimentally validated peptides, aureocyclicin 4185, leucocyclicin Q, and garvicin ML.

### 2.3. Primary Sequence Analysis of Identified Groups in the Sequence Similarity Network

Each group in the sequence similarity network (Figure 2) was analyzed for primary sequence conservation. Amino acid sequences within a cluster that consists of three or more members (i.e., groups 1 to 8) were aligned using Clustal Omega [41], and results were visualized using WebLogo3 [42].

**Figure 3:**
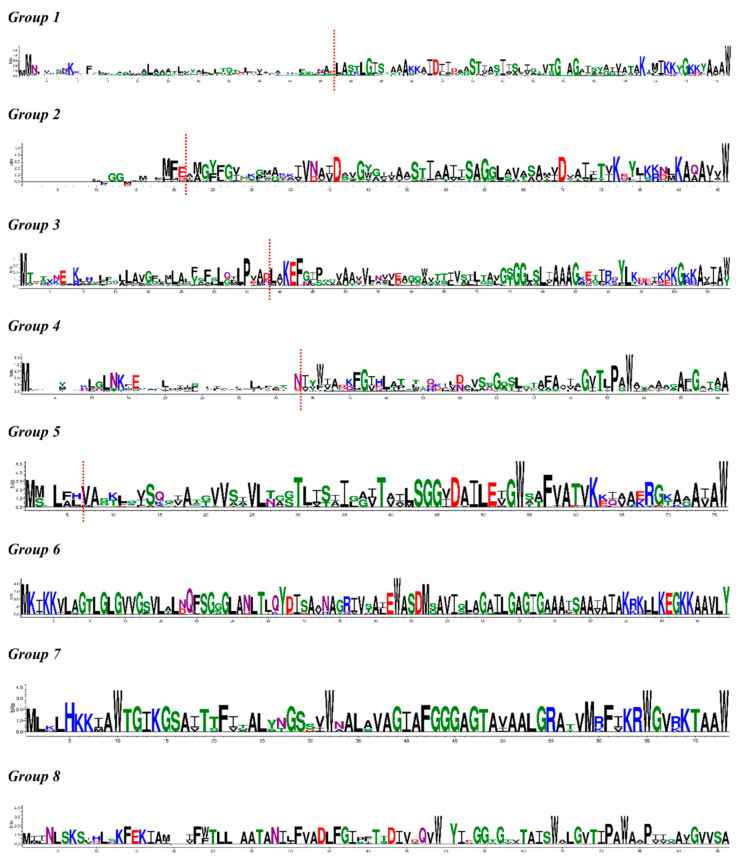
Amino acid sequence logos for precursor peptides of head-to-tail cyclized bacteriocins belonging to groups 1 to 8 of the sequence similarity network in Figure 2. Polar, neutral, basic, acidic, and hydrophobic amino acids are shown in green, purple, blue, red, and black, respectively [42]. The red broken lines indicate the predicted leader peptide cleavage site based on the characterized members of groups 1 to 5. Groups 6 to 8 do not have characterized members, and hence, the cleavage sites could not be proposed.

The sequence logos for groups 1 to 8 (Figure 3) show that the N-terminal region of the precursor peptides displays the most diversity, especially for groups 1 to 4. This region corresponds to the leader sequence that is cleaved during biosynthesis. On the other hand, the peptide sequences of the core peptides (i.e., C-terminus) are more conserved. The mechanism of the cyclization of head-to-tail cyclized bacteriocins is poorly understood, but it is worth noting that the majority of the sequence logos end with an aromatic amino acid (i.e., tryptophan or tyrosine), except for groups 4 and 8 that end in alanine. It appears that a hydrophobic C-terminal amino acid may be an essential requirement for core peptide cyclization.

The currently used classification scheme for head-to-tail cyclized bacteriocins is based on the hydrophobicity and the overall charge of the peptide sequences (i.e., subgroup i peptides are cationic, while subgroup ii peptides are neutral). The sequence logos show that groups 4 and 8 lack positively charged amino acids (i.e., lysine and arginine; colored in blue) and may be regarded as subgroup ii bacteriocins. In addition, members of group 11 and a singleton corresponding to a bacteriocin produced by *Lactobacillus nodensis* DSM 19682 (Table S1) also display hydrophobic core peptide sequences and may thus be classified as subgroup ii head-to-tail cyclized bacteriocins. The rest of the groups and singletons have highly conserved positively charged amino acids in the core peptide region, suggesting that they may be classified as subgroup i head-to-tail cyclized bacteriocins. These positively charge residues are proposed to introduce an overall cationic property to these bacteriocins, thereby facilitating their initial interactions with negatively charged phospholipids in bacterial membranes. Notably, there also appears to be several highly conserved tryptophan residues throughout the core peptide sequences apart from the C-terminal amino acid mentioned earlier. Tryptophan residues are implicated in the anchoring of a bacteriocin to target bacterial membranes through hydrophobic interactions [43].

### 2.4. Identification of the Leader Peptide Cleavage Sites

Based on the experimentally validated head-to-tail cyclized bacteriocins, the lengths of the leader peptides can range from 2 to 48 amino acid residues [7]. The variation in length and composition of the leader sequences complicates our understanding of their potential roles in the biosynthesis of this family of bacteriocins. Out of the eight sequence logos presented in Figure 3, groups 1 to 5 have experimentally confirmed members. Hence, the leader peptide cleavage sites for these groups were deduced based on the amino acid sequences of the characterized members. The cleavage sites are indicated with red broken lines in Figure 3. For other bacteriocin classes (e.g., linear two-peptide bacteriocins) [44], cleavage motifs are established, and hence, the leader peptide cleavage sites are readily identified. However, for the head-to-tail cyclized bacteriocins, our sequence alignment results highlight how challenging it is to postulate how the leader peptide cleavage and core peptide cyclization proceed as there appears to be no apparent sequence motif shared at the cleavage sites across the different groups. Members of group 1 have relatively longer leader sequences, and sequence conservation appears to start only at the first amino acid at the N-terminus of the core peptide. This contrasts with group 2 where there is an evident MFE motif prior to the cleavage site, followed by a series of hydrophobic amino acids at the N-terminus of the core peptide. As for group 3, there is a highly conserved proline residue four amino acids prior to the cleavage site, and similar to group 2, the first few amino acids at the N-terminus of the core peptide are highly conserved, especially the KEF motif for positions 3 to 5 of the core peptide. Group 4, which includes all the currently known subgroup ii head-to-tail cyclized bacteriocins, displays the previously noted highly conserved asparaginyl cleavage site [45]. The N-terminus of the core peptides consists of hydrophobic and aromatic residues. Lastly, group 5 consists of members with short leader peptides (i.e., up to six residues) that are more highly conserved compared to those observed in the earlier groups. The first few residues at the N-terminus of the core peptide are highly conserved.

### 2.5. Biosynthetic Gene Cluster Analysis

**Figure 4:**
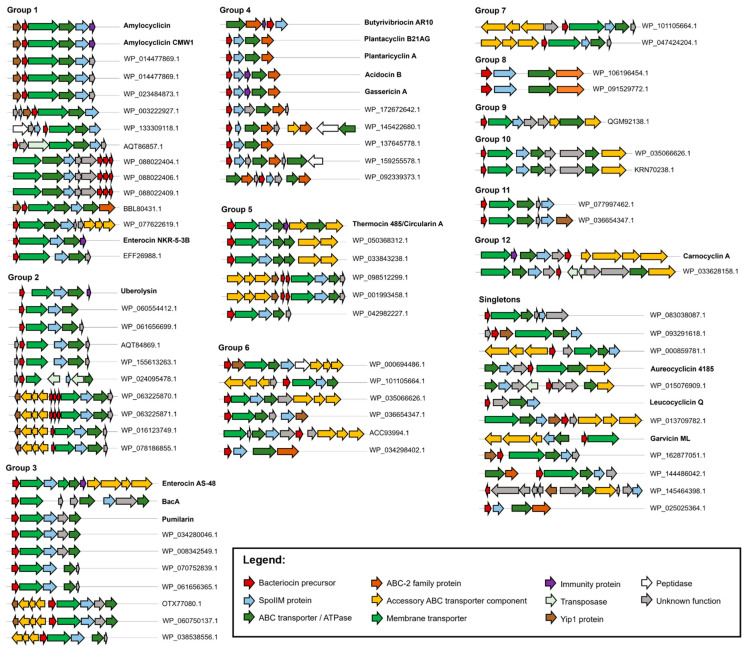
Representative biosynthetic gene clusters of head-to-tail cyclized bacteriocins. Characterized bacteriocins are labelled in bold with the bacteriocin name, while the putative bacteriocins are indicated with their respective protein accession number.

Representative biosynthetic gene clusters of the putative bacteriocins are presented in Figure 4. The gene clusters of all hits identified in this study consist of genes encoding a precursor peptide, a SpoIIM protein, and at least one transporter protein. Interestingly, some gene clusters include multiple copies of the precursor peptide gene, such as the three copies observed for some members of groups 1 and 2. Group 5 also has members with biosynthetic gene clusters comprised of two copies of the precursor peptide gene. The gene organization is mostly conserved (with few variations) among members of the same group except for group 6. Six different gene organizations were observed for this group (Figure 4) among the eight unique precursor peptide sequences that comprise group 6 (Table S1). Group 6 has no characterized member.

Genes encoding transposases that are associated with horizontal gene transfer can be found across the different groups. Another common occurrence is the presence of a Yip1 protein, which is a membrane protein that may be involved in transport. The presence of Yip1 protein is prevalent especially in group 1. Groups 2, 3, 5, and 11 also have members with Yip1 protein, particularly the gene clusters consisting of a set of three genes that are putative accessory ABC transporter components.

One of the longstanding questions about the biosynthesis of head-to-tail cyclized bacteriocins is how the leader peptide is cleaved and the core peptide is cyclized. To date, no enzyme has been identified to facilitate this transformation. It was postulated that genes encoding a peptidase/protease that catalyzes this reaction should be located elsewhere outside the gene cluster [30]. Intriguingly, a few gene clusters encountered in our study contained genes encoding peptidases. These clusters include two members of group 1 wherein a gene encoding a peptidase belonging to the M48 family of zinc peptidases is located three genes downstream from the bacteriocin precursor gene (Figure 4). Another zinc metallopeptidase of the M50 family is found in group 6, located in between a SpoIIM gene and a group of three genes encoding proteins putatively involved in transport (Figure 4). On the other hand, a number of putative peptidases are observed in some gene clusters belonging to group 4, which comprises peptides that are currently classified as subgroup ii head-to-tail cyclized bacteriocins. The possible involvement of these peptidases in leader peptide cleavage and core peptide cyclization remains to be determined experimentally.

### 2.6. Phylogenetic Distribution of Identified Head-to-Tail Cyclized Bacteriocins

**Figure 5:**
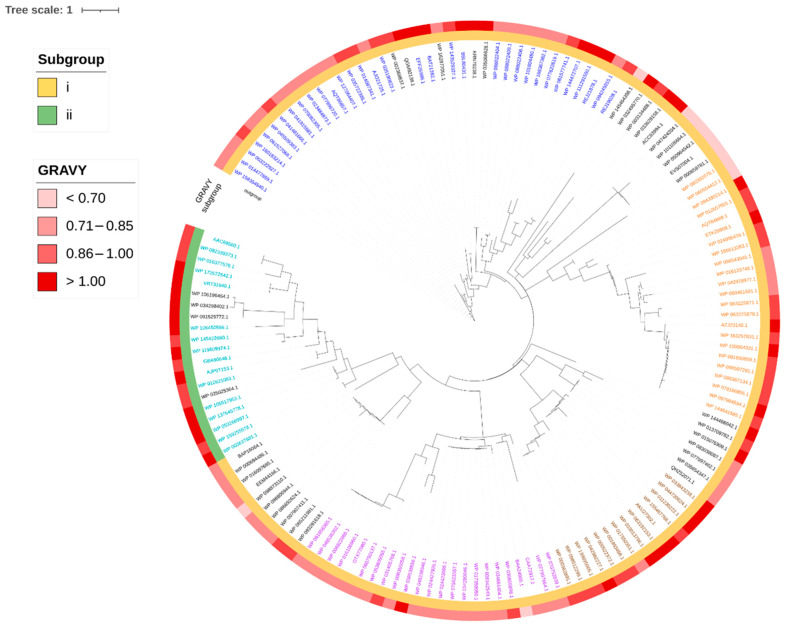
Phylogenetic relationships among putative head-to-tail cyclized bacteriocins identified using the transporter-guided genome mining. The maximum likelihood phylogenetic tree was generated using IQ-TREE [46] and annotated and visualized using iTOL [47]. Members of the five biggest groups in the sequence similarity network analysis are shown in different colors (Group 1—blue, 2—orange, 3—violet, 4—teal, 5—brown). The subgroup number of each accession number and their corresponding hydrophobicity based on GRAVY analysis are indicated in the annotation.

The evolutionary relationship of the head-to-tail cyclized bacteriocins was inferred through phylogenetic analysis. The five biggest groups identified in the sequence similarity network analysis (Figure 2) are shown in the phylogenetic tree in Figure 5.

In the phylogenetic tree, members of groups 1 to 5 are shown in blue, orange, violet, teal, and brown, respectively. The results revealed that group 1, which is the biggest group, evolved mainly from the earliest ancestors of head-to-tail cyclized bacteriocins. This group appears to be highly heterogeneous as the members are distributed into different subclades and exhibit varying degrees of hydrophobicity based on the grand average of hydropathicity index (GRAVY) data. Most of the other groups eventually evolved from a common ancestor that diverged from group 1 early in the evolution. As shown in the phylogenetic tree, the next three biggest groups belonging to subgroup i head-to-tail cyclized bacteriocins; namely groups 2, 3, and 5 form more tightly related subclades, and the hydrophobic character is more conserved among members of each group based on the GRAVY analysis. 

Subgroup ii head-to-tail cyclized bacteriocins, on the other hand, appear to diverge later from subgroup i members. Subgroup ii mainly include groups 4 and 8 members, which are both found to be more closely related to the singleton, leucocyclicin Q, and group 6 members. GRAVY analysis indicates that subgroup ii members appear to be consistently more hydrophobic compared to the members of subgroup i bacteriocins.

## 3. Materials and Methods

### 3.1. Genome Mining for Head-to-Tail Cyclized Bacteriocins

The amino acid sequences of transporter proteins associated with thirteen subgroup i (enterocin AS-48 [48], circularin A [49], uberolysin [50], carnocyclin A [51], enterocin NKR-5-3B [52], pumilarin [29], garvicin ML [53], aureocyclicin 4185 [54], leucocyclicin Q [13], amylocyclicin CMW1 [55], amylocyclicn [56], BacA [57], and thermocin 485 [58]), and six subgroup ii (acidocin B [45], gassericin A [59], butyrovibriocin AR10 [60], plantaricyclin A [61], plantacyclin B21AG [62], and paracyclicin [63]) head-to-tail cyclized bacteriocins were obtained and used as driver sequences to mine the NCBI database for putative transporter proteins associated with head-to-tail cyclized bacteriocins using protein BLAST with default search parameters. Duplicates were removed using Excel processing and the accession numbers were inputted into the RODEO Web Tool [31] to extract the genome neighborhood for the various hits and allow for manual identification of high probability putative bacteriocin gene clusters characterized by the presence of genes encoding a precursor peptide, a SpoIIM protein, in addition to the transporter protein(s).

### 3.2. Precursor Peptide Sequence Similarity Analysis

A list of the precursor peptides from the RODEO analysis was retrieved and a sequence similarity network was generated using the EFI Enzyme Similarity Tool [64,65]. This allowed an all-by-all BLAST analysis (E-value 1 × 10^−5^) and clustered together the most related proteins into groups. To generate the final sequence similarity network, different alignment scores were tested to select an appropriate clustering that grouped most hits with similar gene cluster organization together. This was obtained with a percent identity cut-off of at least 50%. Cytoscape (version 3.9) was then used to visualize the results [38]. The list of peptides with their associated producer organisms is presented in Table S1.

### 3.3. Generation of Precursor Peptide Sequence Logos

Groups from the sequence similarity network that consist of three or more members were subjected to further sequence analysis. The precursor peptide amino acid sequences belonging to each group were aligned using Clustal Omega [41], and the alignment results were used to create sequence logos using WebLogo3 [42]. The leader peptide cleavage sites were then inferred based on the cleavage sites of known head-to-tail cyclized bacteriocins.

### 3.4. Phylogenetic Analysis

Multiple sequence alignment of the putative head-to-tail cyclized precursor peptides was performed using the G-INS-i method implemented in the MAFFT web server (https://mafft.cbrc.jp/alignment/server/) [66]. IQ-TREE web server (http://iqtree.cibiv.univie.ac.at/, accessed on 17 October 2021) [46] was then used to determine the best-fit model for protein alignment (WAG + F + I + G4) based on the Bayesian information criterion score and to construct a phylogenetic tree using the maximum likelihood method. The phylogenetic tree was then visualized and annotated using iTOL v4 [47]. Hydrophobicity analysis was performed using the GRAVY Calculator tool (http://gravy-calculator.de).

## 4. Conclusions

In this study, we performed a new genome mining strategy to search for novel head-to-tail cyclized bacteriocins, specifically by using transporter protein sequences associated with 19 experimentally validated head-to-tail cyclized bacteriocins as query sequences, and the use of a set of bioinformatics tools. A total of 387 strains that potentially encode putative head-to-tail cyclized bacteriocins were identified, 127 of which are unique peptide sequences. Sequence similarity network, sequence analysis, and biosynthetic gene cluster analysis revealed that head-to-tail cyclized bacteriocins are widely distributed and are more diverse than previously thought. Phylogenetic analysis showed that members of group 1 in the sequence similarity network are the most evolutionarily diverse and that the other groups arose from a common ancestor that diverged from group 1. While this work identified putative novel head-to-tail cyclized bacteriocins, it does not predict whether these peptides are easily produced in the laboratory setting and to which pathogens will they be active against. Hence, future work must be directed towards peptide isolation and characterization. It is of particular interest to characterize representative members of groups in the sequence similarity network that do not currently have an experimentally characterized member. Upon the successful production of these bacteriocins, purified peptides can then be used in bioactivity assays against a range of bacterial indicator strains to determine the sensitivity and efficacy of their antimicrobial potential. This work sets the stage for isolation and characterization of diverse head-to-tail cyclized bacteriocins, with likely varying specificities, for their subsequent applications as biopreservatives and antimicrobial agents in various applications in food, animal, and health industries.

## Data Availability

The data presented in this study are available in the supplementary material.

## Supplementary Materials

The following are available online, Table S1: A complete list of head-tail cyclized bacteriocins identified in this study, classified based on the clustering result of the sequence similarity network analysis.
